# Building the Nigerian Palliative Care Workforce: An Interdisciplinary Distance Learning Training Program

**DOI:** 10.5334/aogh.3744

**Published:** 2022-10-27

**Authors:** Ann Ogbenna, Denise Drane, Autumn N. Crowe, Oluwafikewa Oyedele, Joshua Hauser, Olaitan Soyannwo, Adeboye Ogunseitan, Ashti Doobay-Persaud

**Affiliations:** 1 Department of Hematology and Blood Transfusion, Faculty of Clinical Sciences, College of Medicine, University of Lagos, Lagos, NG; 2 Searle Center for Advancing Learning and Teaching, Northwestern University, Evanston, IL, US; 3 Institute for Global Health, Feinberg School of Medicine, Northwestern University, Chicago, IL, US; 4 College of Medicine, University of Lagos, Lagos, NG; 5 Department of Medicine, Division of Hospital Medicine, Section of Palliative Care, Northwestern University Feinberg School of Medicine, Chicago, IL USA; 6 Palliative Care Service, Jesse Brown VA Medical Center, Chicago, IL; 7 Department of Anesthesia, College of Medicine, University of Ibadan, NG; 8 Division of Hospital Medicine, Departments of Medicine and Medical Education, Feinberg School of Medicine, Northwestern University, Chicago, IL 60611, USA

**Keywords:** Palliative Care, Health Care Service Delivery, Global Health Education

## Abstract

**Background:**

Education and capacity building in palliative care are greatly needed in Nigeria. Currently, two institutions integrate palliative care into the undergraduate medical curriculum and no post graduate training exists. A team from the University of Lagos in Nigeria and Northwestern University in the US collaborated to design, implement, and evaluate a 12-hour virtual palliative care training program for Nigerian health professionals.

**Objective:**

This study investigated the impact of the first session of the training program on healthcare professionals’ knowledge, skills, attitudes, and confidence in palliative care.

**Methods:**

The Education in Palliative and End-of-Life (EPEC) curriculum and the Kenya Hospices and Palliative Care Association (KEHPCA) curriculum were used as foundations for the program and adapted for the Nigerian context. Delivered online, the training focused on goals of palliative care, whole patient assessment, communication skills, pain management, psychosocial issues, palliative care in COVID, oncology, and HIV. A mixed-methods evaluation based on Kirkpatrick’s evaluation framework was used and data were gathered from surveys and focus groups.

**Findings:**

Thirty-five health professionals completed the training. The training had a positive impact on knowledge, skills, and attitudes. Confidence in providing end-of-life care increased from 27.3% to 92.9% while confidence in prescribing medication to relieve symptoms at the end of life increased from 42.9% to 92.0%. Performance on multiple-choice knowledge tests increased by 10% (p < 0.01). All participants stated that they would recommend the program to a peer while 96.4% reported the program was relevant to the Nigerian context. Qualitative analysis suggested that the training would help participants provide more holistic care for patients, communicate better, and change how they interacted with families. Topics to be addressed in future training were identified.

**Conclusions:**

This virtual training can be an important element in palliative care capacity building in Nigeria and represents a model for global health collaboration.

## Introduction

The healthcare workforce shortage in palliative care is a global health crisis [1,2]. The global workforce shortage has the greatest impact on low- and lower-middle income countries [3]. Such shortages impact access to healthcare services and contribute to the perpetuation of health inequities. While the global healthcare workforce crisis impacts all disciplines of medical practice, globally there is a paucity of palliative care providers. It is estimated that 40 million individuals need palliative care each year, with 78% of people in need residing in low- and middle-income countries [3]. The World Health Organization (WHO) describes palliative care as: an approach to improve the quality of life of patients and patients’ families who are impacted by life-threatening illnesses through prevention of and relief of pain and suffering and addressing the physical, psychosocial, and spiritual needs of patients and their families [3]. Due to the aging population, an increased life span for those living with chronic conditions and the global increase in the incidence of cancers, there is an unmet need in providing care to these patient populations owing to shortage of providers trained in palliative care. This unmet need may be due to countries’ lacking national health policies regarding the provision of palliative care in addition to the lack of healthcare systems which both employ palliative care providers and train future clinicians in palliative care.

Investigations of palliative care training have found that medical students receive inadequate training in palliative care and that physicians feel unprepared and uncertain about providing palliative care [4,5]. Studies investigating the impact of training in palliative care have demonstrated that training increases the learner’s knowledge about palliative care topics such as symptom management, ethics, and communication skills. Moreover, palliative care training is associated with less aggressive treatment plans in end-of-life care and the adoption of palliative care principles [6,7,8,9].

Based on the WHO’s estimate that 1% of the population requires palliative care, approximately 10 million people in Africa are in need of palliative care [10]. However, palliative care is still in its rudimentary stages in most African countries with only about 26% showing any increases in palliative care service over the last 15 years [11]. A training and education gap also exists across the continent, with few opportunities for formal graduate or postgraduate palliative care training programs [11].

In Nigeria palliative care was introduced in the early 1990’s, however, gained momentum a decade later when it was re-introduced through new policy initiatives with a call in 2008 to establish palliative care services in tertiary health institutions [12,13]. Today, according to the global directory of PC Institutions and Organizations, Nigeria has 10 centers offering palliative care: six through institutions, one through a state program, and three through non-governmental organizations [14].

There is an important opportunity to develop and fill both the informal and formal education gaps for palliative care education in Nigeria. Currently, only two institutions have successfully integrated palliative care into their undergraduate medical curricula and there is no postgraduate training in palliative care [12]. A study by Oyebola in 2017, conducted during the seventh annual HPCAN Conference in Nigeria, showed that even among those currently practicing palliative care, only 47% (8/17) have had any formal palliative care training while, 53% (9/17) have “ad-hoc training post basic.” Other studies have also demonstrated low levels of palliative care knowledge among Nigerian trainees [15].

This current study sought to evaluate the impact of a palliative care training provided to clinical healthcare professional practitioners in Nigeria through a partnership between the University of Lagos and Northwestern University in the US. The partnership seeks to provide and increase access to training in palliative care. Goals of the partnership were to develop and hold three palliative care trainings for health care professionals and to help develop a palliative care center at the University of Lagos. Team members at the University of Lagos, Northwestern University, and other invited palliative care professionals in Nigeria, selected palliative care topics which were adapted for the Nigerian context. This paper describes the first training in palliative care which was held over three days in November 2020 and presents evaluation data on the impact of the training. The training sought to provide participants with foundational (primary) palliative care knowledge and skills [16]. Learning objectives were to provide learners with, (a) the foundational knowledge of the principles, models, and current practices of primary palliative care; (b) information about providing difficult news, diagnosis, and prognosis techniques; (c) skills to assess the psychological, social, and spiritual needs of patients and families facing serious illness and dying, and (d) knowledge in completing a primary palliative care assessment of patients.

## Methods

### Training Program Theory of Change

**Figure 1:**
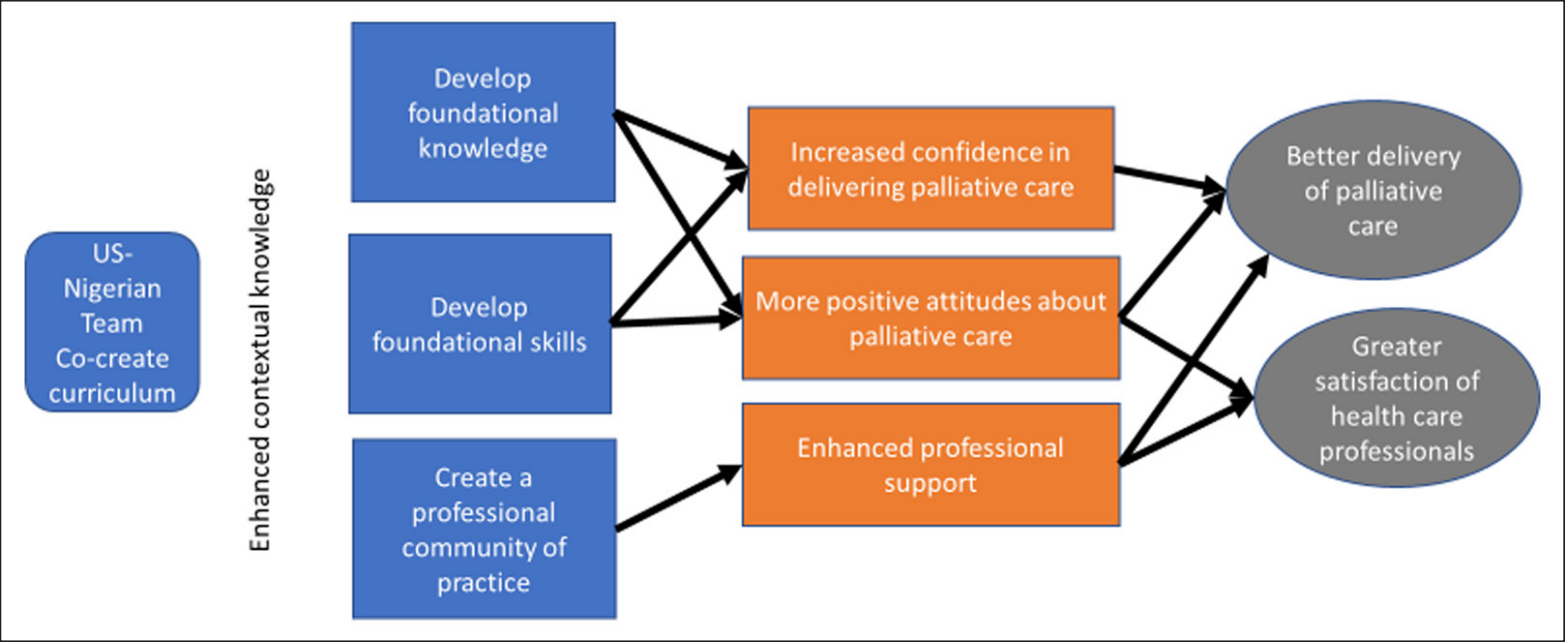
Program theory of change.

The program’s theory of change is depicted in Figure 1. The curriculum was co-created by a team of US and Nigerian palliative care health professionals with the aim of enhancing knowledge of the local context. The program was designed to provide foundational knowledge and skills in palliative care. The assumption was that enhanced knowledge and skills would lead to great professional self-confidence and empowerment in delivering palliative care. In addition, the program was designed to incorporate interactive, small group activities. It was hypothesized that participating in small group interactive activities during the training would enhance learning and create a sense of community which would also lead to greater confidence in providing palliative care.

### Training program description

The training program was co-developed by the Nigeria and US team using the Education in Palliative and End-of-Life (EPEC) curriculum [17] and the National Palliative Care Training Curriculum for HIV&AIDS, Cancer and Other Life Threatening Illnesses from the Kenya Hospices and Palliative Care Association (KEHPCA) [18], as a foundation. A team of 12 interdisciplinary health professionals from LUTH in Nigeria reviewed and checked for the adaptability of the EPEC curriculum to the Nigeria context. The process of adaptation for context involved assembling a curricular review committee at LUTH team. This interdisciplinary committee was selected because of their experience and were asked to use their expertise and relevant Nigerian standards to adapt the curriculum. They were provided with publicly available modules to review: the US based EPEC curriculum, EPEC India (EPEC curriculum adapted by partners in India) and the Kenya Hospices and Palliative Care Association (KEHPCA) Curriculum. Specifically, areas of cultural, legal, spiritual, and historical differences were addressed. Also, areas requiring data specific to Nigeria were also added. Case studies were also adapted to mirror Nigerian standards and context and the videos were also reproduced to reflect Nigerian culture and indigenous knowledge. Overall, the content was framed to allow for cultural sensitivity. Reviewers including invited palliative care instructors from Nigeria adapted the trainers note, the modules and the slides.

Although initial plans were to deliver the training in-person, due to the COVID-19 pandemic, it was delivered virtually using Zoom and a learning management system called Moodle. It consisted of plenary lectures and small group, interactive breakout sessions. Some of the breakout sessions had facilitators and some were designed for independent discussion. The training spanned three days with an average time of four hours per day including the break times. Table 1 below shows the schedule and learning objectives for each session.

**Table 1:** Training schedule and learning objectives.


TOPIC	LEARNING OBJECTIVES

DAY 1	

**Plenary 1** Elements and Models of Palliative Care	Describe concepts of suffering. Define palliative medicine. Understand the elements of palliative medicine. Distinguish between palliative care and hospice.

**Plenary 2** Palliative care in Nigeria	Review the history and challenges of palliative care in Nigeria. Describe the status of palliative care in Nigeria.

**Plenary 3** Legal issues	Describe the legal consensus that has developed for issues in palliative care in Nigeria. Identify which palliative care issues lack legal consensus. Understand common legal myths/pitfalls that can interfere with quality care.

**DAY 2**	

**Plenary 4** COVID and Palliative care	Define the goals of palliative care in the setting of COVID-19. Review ethical principles for providing care in a pandemic. Discuss ethics of balancing protection of healthcare workers with caring for patients. Discuss the symptomatic management of COVID-19 & palliative care.

**Module 1** Communicating bad news	Understand the importance of communication of difficult news as an important and generalizable skill. Apply a six-step protocol for delivering difficult news & clarifying diagnosis & prognosis. Identify difficulties inherent in prognostication.

**Module 2** Goals of care	Identify ways of discussing hope with patients with serious illness to help frame goals of care. Discuss potential goals of care. Use a framework protocol to negotiate goals of care. Identify goals when patient lacks capacity to make decisions.

**Module 3** Ethical Issues in End-of-life care: Medical futility	Identify factors that might lead to situations of futility. Understand how to identify common factors. Describe six steps involved in attempting to resolve conflict in futility situations.

**DAY 3**	

**Plenary 5** Cancer and palliative care	Describe the burden of cancer and cost to society in Nigeria. Describe the role of palliative care in various cancers in Nigeria. Define the settings in which palliative care in cancer can be practiced. Describe the various complications associated with cancers. Describe an approach to maintaining hope amidst the finality of death.

**Plenary 6** HIV and palliative care	Discuss the unique needs of patients with HIV/AIDs & palliative care needs. Apply counseling skills in patients with HIV/AIDS & palliative care needs.

**Module 4** Clinical aspects: Whole patient assessment	Describe concepts of suffering. Identify seven assessment areas during an initial patient encounter. Apply a screening tool to facilitate assessment.

**Module 5** Pain assessment and management	Compare and contrast nociceptive and neuropathic pain. Identify the steps of analgesic management. Calculate the conversion between different opioids. Explain the use of adjuvant analgesic agents. Recognize the adverse effects of analgesics and their management.

**Module 6** Psychosocial issues/working with families	Understand and apply a framework for assessing the psychological, social, & spiritual needs of the patient and family facing serious illness and dying. Demonstrate interventions, including the use of family meetings that can be helpful in optimizing communication with patients and their caregivers.


### Participant Recruitment

Participants were recruited through an announcement on a listserv which was sent to health professionals. The listserv was obtained from the research unit of the College of Medicine, University of Lagos. It consisted of names of health care professionals across Nigeria. This announcement described the training and provided contact information to register for those interested in attending. Nigerian members of the Training Curriculum Committee and Nigerian faculties also recruited participants via email.

### Program Evaluation Design

A member of the US team who is a specialist in program evaluation designed and implemented the evaluation in collaboration with the US/Nigerian team. The Kirkpatrick evaluation framework was used to guide the evaluation [19,20]. The evaluation addressed level 1 (Participant Reaction; satisfaction, perceived value of the training, engagement) and level 2 (Participant Learning; acquisition of knowledge skills and attitudes). Given the short time frame of the evaluation, it was not possible to address Level 3 of the Kirkpatrick framework (Participant Behavior; application of knowledge skills and attitudes). However, as an interim measure, participants were asked to report how, if at all, they intended to apply what they had gained from the training. Level 4 (Results) was also not addressed in this evaluation.

The evaluation consisted of a pre-program survey, pre-post multiple choice knowledge tests, daily surveys focusing on participants’ perceptions of the extent to which session learning outcomes were achieved, and a post-program survey. The pre-program survey collected data on demographic characteristics and asked participants to rate their knowledge, skills, attitudes and confidence in palliative care. Pre-post knowledge test questions were adapted from EPEC to test knowledge related to modules 1–6. Each question consisted of a clinical scenario, followed by a multiple-choice question. Survey questions on learning outcomes were developed in conjunction with the instructors. The post-program survey repeated the same ratings questions from the pre-program survey and included open-ended questions about what participants had gained from the training, how they planned to apply what they had learned, how what they had learned might help them address challenges that they face in their work and unmet training needs. Questions on how well the training addressed considerations related to poverty, children, and the special role of women in Nigeria, were also included.

### Data Collection

Surveys and pre-post knowledge tests were completed electronically via Google forms. For the preregistration survey, participants were sent the survey link via email after they were recruited to attend the training. Participants were given the survey link at the end of each of the three training days for the three post session surveys. Emails with the survey links were also sent to participants to encourage completion of the surveys.

### Data Analysis

Demographic characteristics, rating scale data and knowledge test scores were analyzed using descriptive statistics. Responses to pre and post surveys and pre-post knowledge tests were compared using paired t-tests. Analyses were conducted using SPSS 27.0 for Windows. Open-ended questions were analyzed using thematic analysis.

## Results

### Characteristics of participants

Thirty-five people participated in training (Table 2). Most participants (74.3%) were female and were from teaching hospitals (86.6%). The majority were physicians (40%), followed by nurses (28.6%). Most (70.0%) participants had no previous training in palliative care and served both children and adults.

**Table 2:** Characteristics of participants (n = 35).


CHARACTERISTIC	FREQUENCY	PERCENT

Gender		

Male	9	25.7

Female	26	74.3

Institution		

University of Lagos	21	60.0

University of Ibadan, University College Hospital	7	20.0

University of Jos Teaching Hospital	3	8.6

College of Medicine University of Lagos	1	2.9

Lagos Island Maternity Hospital	1	2.9

Neuropsychiatric Hospital Calabar	1	2.9

Private Hospitals	1	2.9

Profession		

Physicians	14	40.0

Nurse	10	28.6

Pharmacist	8	22.9

Allied Health Professional	2	5.7

Psychologist	1	2.9

Specialty Physicians		

Community Health and Primary Care	1	2.9

Family Medicine	2	5.7

General Practitioner	2	5.7

HIV Medicine	2	5.7

Medical Officer	1	2.9

Obstetrics and gynecology	3	8.6

Palliative Care	1	2.9

Psychiatry	2	5.7

Nurse		

Caregiver	1	2.9

Community Health Nurse Practitioner	1	2.9

Critical care/Infection Prevention and Control	1	2.9

Medical-Surgical Nurse	1	2.9

Midwifery	1	2.9

Nurse clinician	1	2.9

Nurse Educator	1	2.9

Oncology Nurse	2	5.7

Public Health Nurse	1	2.9

Clinical pharmacist	8	22.9

Allied Health Professional		

Acute and critical care physiotherapist	1	2.9

Neurological physiotherapist	1	2.9

Clinical Psychologist	1	2.9

Primary Work Setting		

Teaching Hospital	30	85.7

General Hospital	2	5.7

Private Hospital	2	5.7

Hospice and/or Palliative Care Program	1	2.9

Local of Practice		

Suburban (Town)	1	2.9

Urban	34	97.1

Clinical Population Served		

Adults	9	25.7

Children and Adults	25	71.4

Children, Adults, and Adolescents	1	2.9

Previous Training in Palliative Care		

No	26	74.3

Yes	9	25.7


### Appropriateness and relevance of training to the Nigerian context

Over 95% of the participants felt the training was relevant to their work with 60.3% strongly agreeing. Thirty-one (96.4%) agreed/strongly agreed that the training was culturally appropriate for the Nigerian setting (Table 3A). Participants agreed that the training addressed the following special needs in Nigeria quite well or very well in the following order, poverty (64.3%), children (53.6%), and special roles of women (42.9%) (Table 3B).

**Table 3A:** Participant ratings of appropriateness and relevance of training.


	STRONGLY AGREE	AGREE	NEUTRAL	DISAGREE	STRONGLY DISAGREE

Relevance to the context that the respondent works in					

Day 1	21 (53.8%)	17 (43.6%)	1 (2.6%)	0 (0.0%)	0 (0.0%)

Day 2	16 (66.7%)	8 (33.3%)	0 (0.0%)	0 (0.0%)	0 (0.0%)

Average score	19 (60.3%)	13 (38.5)	1 (2.6%)	0 (0.0%)	0 (0.0%)

Appropriateness to the cultural context in Nigeria					

Day 1	16 (41.0%)	21 (53.8%)	1 (2.6%)	1 (2.6%)	0 (0.0%)

Day 2	13 (54.2%)	11 (45.8%)	0 (0.0%)	0 (0.0%)	0 (0.0%)

Average	15 (46.6)	16 (49.8 %)	1 (2.6%)	1 (2.6%)	0 (0.0%)


**Table 3B:** Participant ratings of how well the training addressed special considerations in Nigeria (n = 28).


	RATING (% OF RESPONDENTS)			

	NOT WELL AT ALL	NOT SO WELL	QUITE WELL	VERY WELL

**The special role of women in Nigeria**	39.3%	17.9%	28.6%	14.3%

**Poverty**	25.0%	10.7%	46.4%	17.9%

**Children**	35.7%	10.7%	50.0%	3.6%


### Daily surveys about achievement of learning objectives

**Figure 2:**
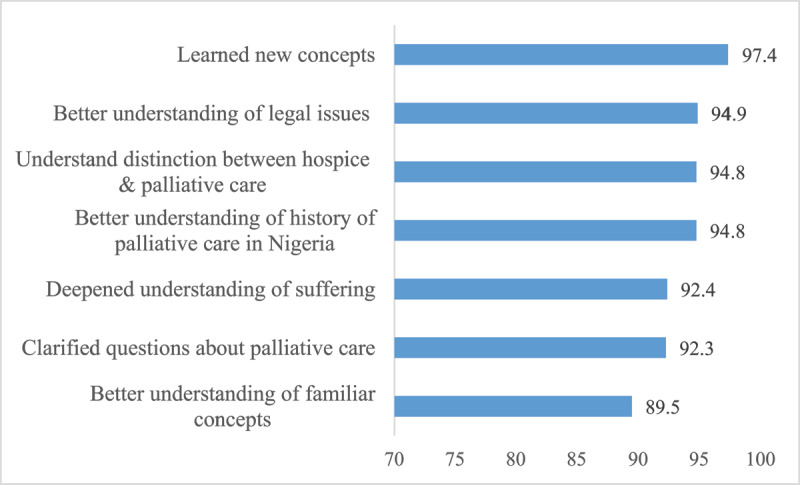
Percent of participants who agreed or strongly agreed that Day 1 learning objectives had been met.

**Figure 3:**
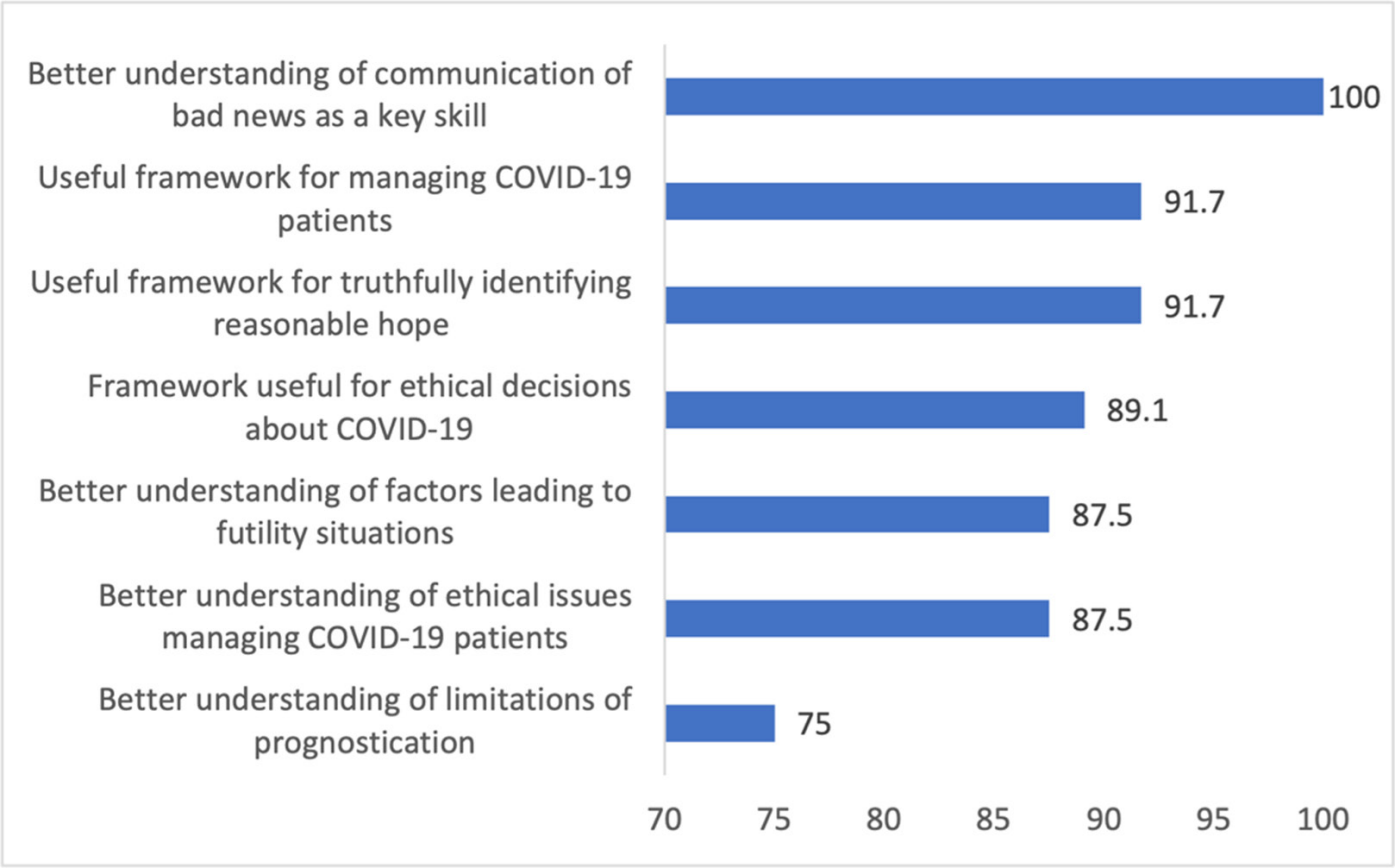
Percent of participants who agreed or strongly agreed that Day 2 learning objective had been met.

Figures 2 and 3 show participants’ ratings of whether the learning objectives for the day was met. There was strong agreement that the Day 1 learning objectives had been met, with over 90% of participants agreeing or strongly agreeing that the training enhanced their understanding of the concepts of palliative care, increased their ability to distinguish between hospice care and palliative care, and provided a better understanding of the legal issues and history of palliative care in Nigeria (Figure 2).

A similar pattern was observed in Day 2 post-training survey results with more than 90% of participants reporting that they had a better understanding of the concepts taught (Figure 3). Participants ratings for learning objectives related to COVID-19 were slightly lower, but still approached 90%. The only exception was the learning objective related to the understanding of the limitations of current knowledge about prognostication with 75% of participants agreeing or strongly agreeing.

### Impact of Training on Palliative Care Knowledge and Skills

Twenty-seven respondents (96.5%) indicated that the training was extremely useful or very useful for their professional practice. Twenty-six (92.8%) reported that they were now extremely or very confident in providing end-of-life care.

The end of training survey findings were positive with almost 100% of participants agreeing, or strongly agreeing that they had benefitted from the training. Sixty-five percent of participants strongly agreed that the training gave them a clearer understanding of the goals of palliative care and helped them consolidate and expand existing knowledge in palliative care (Table 4). Some 60% of the participants strongly agreed that they felt more empowered as a healthcare professional to communicate and 53.6% agreed that they felt more confident because of the training. There was also strong agreement among participants that they had gained in communication skills with almost 70% strongly agreeing that they felt more comfortable talking with patients and their families and with other health professionals involved in palliative care. Finally, over 60% of participants strongly agreed that they felt more comfortable supporting dying patients and their families, and that they would be better able to deal with their own emotions associated with providing palliative care.

**Table 4:** Survey data on the impact of training on palliative care knowledge and skills.


	AGREE STRONGLY	AGREE	NEITHER AGREE OR DISAGREE	DISAGREE	STRONGLY DISAGREE

Clearer understanding of the goals of palliative care	71.4%	28.6%	0.0%	0.0%	0.0%

Expanded knowledge in palliative care	64.3%	35.7%	0.0%	0.0%	0.0%

Training helped to consolidate existing knowledge about palliative care	60.7%	35.7%	3.6%	0.0%	0.0%

Feel more empowered as a health care professional as a result of the training	57.1%	42.9%	0.0%	0.0%	0.0%

Feel more self-confident as a result of the training	53.6%	46.4%	0.0%	0.0%	0.0%

More comfortable communicating with patients and their families	67.9%	32.1%	0.0%	0.0%	0.0%

More confident communicating with other health professionals who are involved in providing palliative care	67.9%	32.1%	0.0%	0.0%	0.0%

More confident in making decisions about how to manage patient care	57.1%	39.3%	3.6%	0.0%	0.0%

More comfortable in supporting dying patients and their families	64.3%	35.7%	0.0%	0.0%	0.0%

I will be better able to deal with my own emotions related to providing palliative care	67.9%	32.1%	0.0%	0.0%	0.0%

I would recommend this training to a colleague	78.6%	21.4%	0.0%	0.0%	0.0%


**Figure 4:**
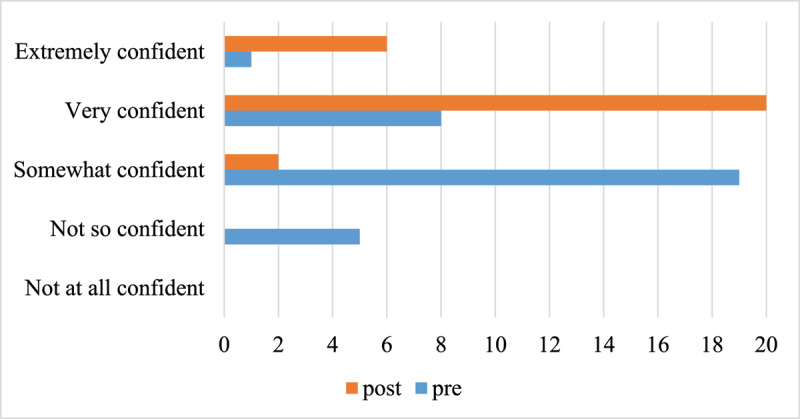
Pre- and post-training ratings of confidence in providing end of life care (X axis displays number of participants).

Participants reported that they gained confidence in providing end of life care during the training. Figure 4 shows a comparison between confidence levels pre- and post-training. Prior to training, most participants (57.6%) reported that they felt somewhat confident with only 27.3% of participants feeling very or extremely confident. Following training, all but two participants (92.9%) reported that they were very confident or extremely confident in providing end of life care.

### Impact on comfort prescribing medicines for symptom relief at the end of life

**Figure 5:**
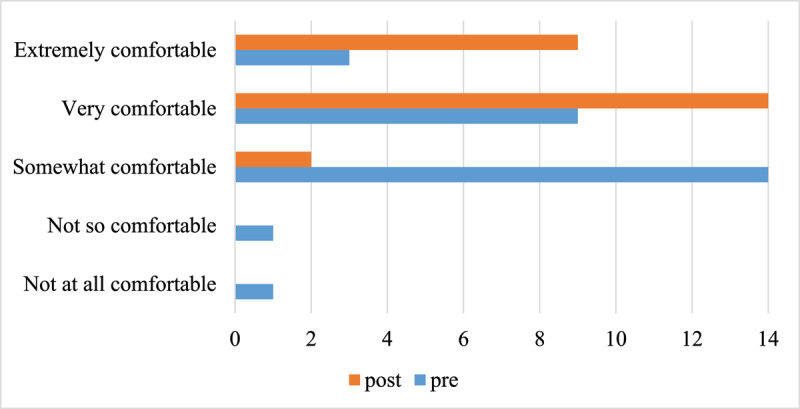
Pre- and post-program survey ratings of comfort in prescribing medicines to manage symptoms. (X axis displays number of participants).

Participants’ comfort in prescribing medicines to manage symptoms at the end of life increased during the training (Figure 5). In the pre-program survey 42.9% of respondents for whom the question was applicable, were very or extremely comfortable compared to 92.0% at the end of the training.

### Knowledge Test Scores Gains

Pre and post module knowledge scores are shown in Table 5. Participants made small gains on both Day 2 and Day 3 tests with scores increasing by approximately 10 percentage points. These differences were statistically significant (Day 2 t = 4.07, df = 19, p = 0.001; Day3: t = 3.53, df = 25, p = 0.002).

**Table 5:** Pre and post knowledge test scores (n = 20).


MODULES	PRE-PROGRAM MEAN % CORRECT (S.D)	POST-PROGRAM MEAN % CORRECT (S.D)	p-VALUE^a^

Day 2: Modules 1, 2, 3 (12 questions)Communication, Goals of Care, Ethical Issues	55.2% (12.3)	66.7% (12.7)	0.001

Day 3: Modules 4, 5, 6 (8 questions)Whole Patient Care, Pain, Psychosocial Issues	46.6% (17.5)	57.2% (15.5)	0.002


^a^ Mean pre and percentage correct scores were compared using paired t-test.

### Qualitative Data Analysis

We analyzed participant responses to open-ended questions about how the training would impact their professional practice and enable them to address challenges associated with palliative care. Participants reported having a greater sense of empowerment and confidence. Consistent with learning objectives, participants reported that they would be able to provide holistic care of patients and would be more effective communicators, being better able to share bad news and better able to explore patients’ understanding of their condition before developing goals. The themes that emerged from the analysis and illustrative quotes are presented in Table 6 below.

**Table 6:** Participants’ responses to open-ended questions on how the training would impact their professional practice and enable them to address challenges associated with palliative care.


THEME	ILLUSTRATIVE QUOTES

**Will be able to provide more holistic care**	“To treat the whole patient and not his/her disease only.” “You don’t treat the patient alone but must consider the totality of the patient. That is, physical, psychological, social, spiritual, etc. of the patient.”

**Gained important communication skills**	“It gives me more self confidence in handling palliative care as I will put what I have learned into practice and those tasks that I usually shy away from like breaking unpleasant news can now be done professionally.” “Adoption of the new lessons learnt will be of great help. For example, exploring what the patient knows and understands about his condition before forming goals of care with the patient.”

**Changes in how they will interact with family members**	“In my area of specialty which is oncology the most common problem is issue of fund for treatment it is a relief that the patient does not have to bear this burden alone but can involve the family members.”

**Increased knowledge about palliative care**	“I have more information now about palliative care. The major challenge for me was lack of information especially in the local context.”

**Increased confidence and sense of empowerment**	“The understanding gained during the training, has empowered me to face any palliative care challenge that may arise.” “This training has made me more confident, enlightened and added to my knowledge on patient care.”

**The need to take a team-based approach to palliative care**	“That collaboration or multidisciplinary teamwork is an integral part [of palliative care].”

**Increased awareness about the gaps in service in Palliative Care in Nigeria**	“It has opened my eyes to see the huge gap in the practice of palliative care in Nigeria. I am now better informed and knowledgeable regarding practice of palliative care which has been lacking in my practice.”


### Unmet needs in Palliative Care Training

Participants identified several unmet needs in palliative care (Table 7). These spanned areas such as palliative care infrastructure, policies, the inclusion of a wider array of medical conditions in future training, and drug administration.

**Table 7:** Unmet training needs reported by participants.


Access to palliative care infrastructure and medicines

Consideration for we the healthcare worker to remain fit before, during and after the unexpected occurs

Government policy on health support (especially financially)

How to effectively encourage patients to accept palliative care

A pharmacist to address issues such as drug-drug and drug-disease aspects of using medications during palliative care, as well as the importance of timing in drug administration

Inclusion of conditions other than cancer and HIV e.g. neurologically impaired chronic low back pain

Nutrition in palliative care

Palliative care for indigent patients

Palliative care for the pediatric age group

Physician interactions


## Discussion

We developed and delivered a three-day online introductory training program in palliative care designed specifically for health care professionals in Nigeria. Our quantitative and qualitative evaluation data suggest that the program improved participants’ foundational palliative care knowledge and increased their self-confidence in delivering palliative care services. Participants reported that they felt better equipped to communicate with patients and their families and to provide holistic care for their patients. Participants who prescribe medication also reported increased confidence in prescribing pain medication to manage symptoms at the end of life. In addition to gaining skills and knowledge, participants also reported benefits of the training at a personal level, feeling more empowered as health professionals, and better able to deal with their own emotions and engage in self-care because of the training. Similar responses to palliative care trainings have been demonstrated in several studies. Artioli et al. (2019) demonstrated the relevance of palliative care training among healthcare professionals in Italy [21]. Similar reports have been documented across the continent particularly in East Africa [22,23,24]. Over 95% of participants felt that the training was appropriate and relevant to their work in Nigeria. This suggests that we were successful in adapting the Education in Palliative and End of Life Care (EPEC) and the Kenya Hospices and Palliative Care Association (KEHPCA) curricula for the needs of Nigerian health professionals working in urban contexts. These results also suggest that a team consisting of a combination of Nigerian and US health professionals was able to create an effective educational initiative. Rawlinson et al. (2014) have highlighted the potential for combining global resources and having stakeholders work together to create educational resources that optimize the understanding of the context in which services are to be delivered and hence optimize the educational outcomes [22].

Although the training was originally planned to be delivered in-person, due to COVID-19 restrictions, the training was successfully implemented online via Zoom on mobile devices such as cell phones. This study adds to the growing body of evidence highlighting the potential for e-learning to deliver quality instruction in palliative care in Nigeria.

An obvious limitation of this evaluation is its focus on largely self-reported gains. We did supplement self-reported measures with some performance measures on multiple-choice test questions. Participants made small, statistically significant, gains that were on the order of 10 percentage points. These gains were disappointing in that average post percent correct scores were below 75%. However, this may have been due to the post-tests being done at the very end of the day when participants were short of time, and because passing the daily knowledge tests was not a requirement for continued participation in the training. It is also important to note that because the training was providing basic, introductory level knowledge of palliative care, the learning outcomes focused on lower levels of Blooms taxonomy of learning objectives: understanding, identifying recognizing, knowing [25].

Another limitation of the evaluation is its focus on short-term outcomes. As the surveys were conducted immediately after the training, participants did not have the opportunity to report how the training impacted their clinical practice. Data from responses to open-ended survey questions certainly suggested that participants could see many opportunities to apply what they had learned e.g., communicating bad news, providing more holistic care, assessing patients’ understanding of their condition before formulating goals, and engaging in multidisciplinary teamwork. We will conduct a long-term follow-up with patients to see if these anticipated changes in practice are carried out.

This basic introductory training has the potential for trainee overconfidence in knowledge acquisition. Following the training, respondents’ confidence levels increased from the 20% range to the 90% range. This increase in confidence was much higher than the post training knowledge assessment which increased to 75% on average. This implies an overconfidence bias that may be present in didactic training sessions not complemented by practice, mentorship or oversight. To deepen learning and teaching and mitigate this outcome, we have created a community of practice platform with the goal of continuous education, feedback and mentoring for participants.

Despite these limitations, this study has shown that we were able to implement a focused virtual training course in palliative care with positive self-reported outcomes. The need for PC training in Nigeria was further corroborated in this study as 74.3% [26] of the participants had had no previous training in palliative care. Several other studies done in Nigeria have shown that the knowledge of palliative care amongst medical practitioners needs great improvement [12,15,26]. Our next steps will now be to support continuous educational capacity building, build a community of practice with participants and facilitate the integration of palliative care services in the Lagos University Teaching Hospital.

## Institutional Review Board Approval

The study was reviewed by the institutional review board of Northwestern University, Evanston, IL and was assigned a determination of Not Human Research. (STU# 00214815).

## References

[1] Powell R, Mwangi-Powell F. Improving palliative care in Africa. BMJ. 2008; 337(23). DOI: 10.1136/bmj.a1566 18812370

[2] Liu JX, Goryakin Y, Maeda A, Bruckner T, Scheffler R. Global health workforce labor market projections for 2030 [published correction appears in Hum Resour Health. 2017 Feb 20;15(1):18]. Hum Resour Health. 2017; 15(1): 11. DOI: 10.1186/s12960-017-0187-2 28159017 PMC5291995

[3] World Health Organization. Palliative Care. https://www.who.int/news-room/fact-sheets/detail/palliative-care. Published August 5, 2020. Accessed December 6, 2021.

[4] Piili RP, Lehto JT, Luukkaala T, Hinkka H, Kellokumpu-Lehtinen PI. Does special education in palliative medicine make a difference in end-of-life decision-making? BMC Palliat Care. 2018; 17(1): 94. DOI: 10.1186/s12904-018-0349-6 30021586 PMC6052558

[5] Pieters J, Dolmans DHJM, Verstegen DML, Warmenhoven FC, Courtens AM, van den Beuken-van Everdingen MHJ. Palliative care education in the undergraduate medical curricula: students’ views on the importance of, their confidence in, and knowledge of palliative care. BMC Palliat Care. 2019; 18(1): 72. DOI: 10.1186/s12904-019-0458-x 31455326 PMC6712798

[6] Adriaansen M, van Achterberg T. The content and effects of palliative care courses for nurses: a literature review. Int J Nurs Stud. 2008; 45(3): 471–485. DOI: 10.1016/j.ijnurstu.2007.01.016 17509596

[7] Fortin Magaña M, Diaz S, Salazar-Colocho P, Feng A, López-Saca M. Long-term effects of an undergraduate palliative care course: a prospective cohort study in El Salvador [published online ahead of print, 2020 Nov 20]. BMJ Support Palliat Care; 2020. DOI: 10.1136/bmjspcare-2020-002311 33219104

[8] Lam PL, Lam TC, Choi CW, Lee AW, Yuen KK, Leung TW. The impact of palliative care training for oncologists and integrative palliative service in a public-funded hospital cluster-a retrospective cohort study. Support Care Cancer. 2018; 26(5): 1393–1399. DOI: 10.1007/s00520-017-3963-6 29138955

[9] Chang J, Qi Z, Jiang S, Li L, Sun Q. The impact of palliative care education and training program on the resident physicians. Ann Palliat Med. 2021; 10(3): 2758–2765. DOI: 10.21037/apm-20-1625 33549004

[10] African Palliative Care Association. Palliative care in Africa: the need. https://www.africanpalliativecare.org/awareness/palliative-care-in-africa-the-need/. Published 2018. Accessed December 6, 2021.

[11] Rhee JY, Garralda E, Torrado C, et al. Palliative care in Africa: a scoping review from 2005–16. Lancet Oncol. 2017; 18(9): e522–e531. DOI: 10.1016/S1470-2045(17)30420-5 28884701

[12] Oyebola FO. Palliative Care Trends and Challenges in Nigeria–The Journey So Far. Emerg Intern Med. 2017; 1(2): 17. http://www.imedpub.com/emergency-and-internal-medicine/.

[13] Soyannwo OA. Palliative care and nursing. Afr J Med Med Sci. 2009; 38(2): 67–70. https://pubmed.ncbi.nlm.nih.gov/20229741/. 20229741

[14] International Association for Hospice & Palliative Care. Global Directory of Palliative Care Institutions and Organizations. https://hospicecare.com/global-directory-of-providers-organizations/. Updated January 15, 2022. Accessed January 16, 2022.

[15] Nnadi DC, Singh S. Knowledge of palliative care among medical interns in a tertiary health institution in Northwestern Nigeria. Indian J Palliat Care. 2016; 22(3): 343–347. DOI: 10.4103/0973-1075.185080 27559266 PMC4973498

[16] Quill TE, Abernethy AP. Generalist plus specialist palliative care—creating a more sustainable model. N Engl J Med. 2013; 368(13): 1173–1175. DOI: 10.1056/NEJMp1215620 23465068

[17] Hauser JM, Preodor M, Roman E, Jarvis DM, Emanuel L. The evolution and dissemination of the education in palliative and end-of-life care program. J Palliat Med. 2015; 18(9): 765–770. DOI: 10.1089/jpm.2014.0396 26302426

[18] Kenya Hospices and Palliative Care Association. National Palliative Care Training Curriculum for HIV&AIDS, Cancer and Other Life Threatening Illnesses. Nairobi, Kenya: Ministry of Health; 2013.

[19] Kirkpatrick DL. Evaluating Training Programs: The Four Levels. San Francisco, CA: Berrette-Koehler; 1994.

[20] Kirkpatrick D. Great ideas revisited. Techniques for evaluating training programs. Revisiting Kirkpatrick’s four-level model. Train Dev. 1996; 50(1): 54–59. https://eric.ed.gov/?id=EJ515660. Accessed January 17, 2022.

[21] Artioli G, Bedini G, Bertocchi E, et al. Palliative care training addressed to hospital healthcare professionals by palliative care specialists: a mixed-method evaluation. BMC Palliat Care. 2019; 18(1): 88. DOI: 10.1186/s12904-019-0476-8 31655585 PMC6815393

[22] Rawlinson F, Gwyther L, Kiyange F, Luyirika E, Meiring M, Downing J. The current situation in education and training of health-care professionals across Africa to optimise the delivery of palliative care for cancer patients. Ecancer medical science. 2014; 8: 492. DOI: 10.3332/ecancer.2014.492 25624873 PMC4303614

[23] Downing J, Kawuma E. The impact of a modular HIV/AIDS palliative care education programme in rural Uganda. Int J Palliat Nurs. 2008; 14(11): 560–568. DOI: 10.12968/ijpn.2008.14.11.31761 19060807

[24] LaVigne AW, Gaolebale B, Maifale-Mburu G, et al. Strengthening palliative care delivery in developing countries: a training workshop model in Botswana. Ann Palliat Med. 2018; 7(4): 444–448. DOI: 10.21037/apm.2018.05.14 30180726

[25] Bloom BS. Taxonomy of Educational Objectives; The Classification of Educational Goals. New York: Longmans, Green; 1956.

[26] Fadare JO, Obimakinde AM, Afolayan JM, Popoola SO, Aduloju T, Adegun PT. Healthcare workers knowledge and attitude toward palliative care in an emerging tertiary centre in South-west Nigeria. Indian J Palliat Care. 2014; 20(1): 1–5. DOI: 10.4103/0973-1075.125547 24600175 PMC3931235

